# Corrigendum to “Polybrominated methoxy- and hydroxynaphthalenes” [Turkish Journal of Chemistry 40 (2) 2016 332–346 ]

**DOI:** 10.55730/1300-0527.3689

**Published:** 2024-08-20

**Authors:** Kıymet BERKİL AKAR, Osman ÇAKMAK, Tuncay TUNÇ

**Affiliations:** 1 Department of Bioengineering, Faculty of Engineering and Natural Science, Gaziosmanpaşa University, Tokat, Turkiye; 2 Department of Chemistry, Faculty of Arts and Science, Yıldız Technical University, İstanbul, Turkiye; 3 Department of Science Education, Faculty of Education, Aksaray University, Aksaray, Turkiye

This corrigendum is to address an issue regarding the manuscript’s previous publication. The authors have regrettably noticed that the incorrect version of the figure and crystal data was used in the original published version of this paper. To rectify this oversight and ensure the accuracy of the published work, the correct figure and data are included for your reference

A single crystal of **6** was obtained by slow evaporation from MeOH enantiomers that crystallizes in the orthorhombic space group *P*2_1_2_1_2_1_ with *Z* = 4. There is one molecule in the asymmetric unit.

## X-ray Crystallography

For the crystal structure determination, single-crystal of the compound 2,3,5,8-tetrabromo-4-methoxy-1,2,3,4-tetrahydronaphthalen-1-ol (**6)** was used for data collection on a Bruker APEX-II CCD diffractometer (equipped with a two-dimensional area CCD detector). Graphite-monochromated Mo-K**_α_** radiation (λ= 0.71073 Å) and oscillation scan technique with Δw = 5° for one image were used for data collection. The lattice parameters were determined by the least-squares methods on the basis of all reflections with *F*^2^>2σ**(***F*^2^ ). The integration of the intensities, correction for Lorentz and polarization effects and cell refinement was performed using Bruker SAINT (Bruker AXS Inc., 2012) software ^9^. The structures were solved by direct methods using SHELXS- 97^10^ allowed for the location of most of the heaviest atoms, with the remaining non-hydrogen atoms being located from different Fourier maps calculated from successive full-matrix least squares refinement cycles on *F*^2^ using SHELXL-97^10^. All non-hydrogen atoms were refined using anisotropic displacement parameters. Hydrogens attached to carbons were located at their geometric positions using appropriate HFIX instructions in SHELXL. The final difference Fourier maps showed no peaks of chemical significance. Crystal data for **6**: C_11_H_10_Br_4_O_2_, crystal system, space group: orthorhombic, *P*2_1_2_1_2_1_; (no:19); unit cell dimensions: a =10.465(3), b=10.620(4), c=12.270(5) Å, α= 90, *β*= 90, γ= 90**°**; volume; 1363.7(9) Å^3^, formula weight ; 493.79, Z = 4; calculated density: 2.405 g/cm^3^ ; absorption coefficient: 11.791 mm^−1^ ; *F* (000): 928; θ-range for data collection 2.3–26.4 °; refinement method: full matrix least- square on *F*^2^; data/parameters: 2792/155; goodness-of-fit on *F*^2^: 1.211; final R-indices [ I > 2 σ(I) ]: R_1_ = 0.051, wR_2_ = 0.083; largest diff. peak and hole: 0.425 and −0.462 e Å^−3^.

CCDC- 2271383 number contains the supplementary crystallographic data for compound **6**. These data are provided free of charge via the joint CCDC/FIZ Karlsruhe deposition service www.ccdc.cam.ac.uk/structures.[Fig f1-tjc-48-04-701]

## Figures and Tables

**Figure. A f1-tjc-48-04-701:**
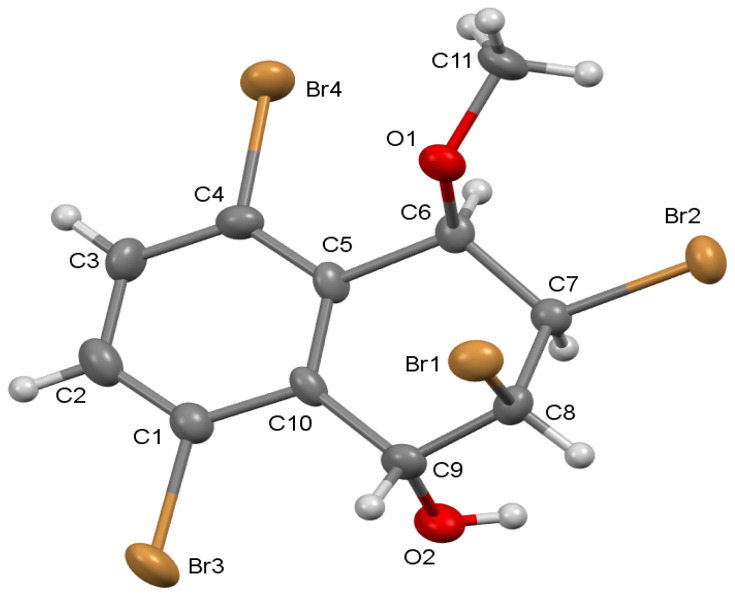
view of the racemate molecule **6**, with the atom-numbering scheme. Displacement ellipsoids are drawn at the 40% probability level. H atoms are shown as small spheres of arbitrary radii.

